# A Mind-inspired Architecture for Adaptive HRI

**DOI:** 10.1007/s12369-022-00897-8

**Published:** 2022-07-25

**Authors:** Alessandro Umbrico, Riccardo De Benedictis, Francesca Fracasso, Amedeo Cesta, Andrea Orlandini, Gabriella Cortellessa

**Affiliations:** ISTC-CNR - Institute of Cognitive Sciences and Technologies, National Research Council of Italy, Via S. Martino della Battaglia 44, Rome, Italy

**Keywords:** Assistant Robotics, Mind-inspired architectures for social robots, Engaging and personalized HRI, Multi-modal interaction

## Abstract

One of the main challenges of social robots concerns the ability to guarantee robust, contextualized and intelligent behavior capable of supporting continuous and personalized interaction with different users over time. This implies that robot behaviors should consider the specificity of a person (e.g., personality, preferences, assistive needs), the social context as well as the dynamics of the interaction. Ideally, robots should have a “mind" to properly interact in real social environments allowing them to continuously adapt and exhibit engaging behaviors. The authors’ long-term research goal is to create an advanced mind-inspired system capable of supporting multiple assistance scenarios fostering personalization of robot’s behavior. This article introduces the idea of a dual process-inspired cognitive architecture that integrates two reasoning layers working on different time scales and making decisions over different temporal horizons. The general goal is also to support an empathetic relationship with the user through a multi-modal interaction inclusive of verbal and non-verbal expressions based on the emotional-cognitive profile of the person. The architecture is exemplified on a cognitive stimulation domain where some experiments show personalization capabilities of the approach as well as the joint work of the two layers. In particular, a feasibility assessment shows the customization of robot behaviors and the adaptation of robot interactions to the online detected state of a user. Usability sessions were performed in laboratory settings involving 10 healthy participants to assess the user interaction and the robot’s dialogue performance.

## Introduction

According to the 2021 Aging Report [27] the EU’s demographic old-age dependency ratio (i.e., the ratio between people aged 65 years and over and those aged 20-64) is projected to increase significantly in the coming decades. Most seniors also want to *age in place* [5], meaning they aim to live independently in their own home for as long as possible. However, older people experience numerous barriers to healthy and active aging also considering the inadequacy of health resources and the scarcity of social supports. By 2030, the World Health Organization (WHO) foresees a worldwide workforce shortfall of healthcare professionals, with dramatic consequences for patients, economies and communities. In this scenario, research is investigating the possible role of technology in general and, more particularly of robots, as a help tool to bridge this gap between the demand for assistance and the actual offer. Mois et al. [40] present an analysis from the point of view of physical, cognitive and social support, highlighting the relevance of assistive robots to facilitate active and healthy aging.

Social Robotics is a growing interdisciplinary research field aiming at supporting humans in different social situations through robot-based solutions. Socially Assistive Robotics (SAR) can be seen as a specific area of Social Robotics focused on supporting humans in assistive scenarios [20, 57] and on assessing their impact and acceptance in realistic scenarios [6]. Results in this field could play a central role to compensate the foreseen workforce shortage and growing assistance demand. Examples of applications concern the use of interacting robots to improve the lifestyle of people with dementia and support their caregivers. In [9] a social robot is integrated with a network of sensors to measure Activity of Daily Living with the aim of providing context-relevant suggestions to the person with dementia and reducing the burden of the caregiver. Recent interests focus on the development of robots that also contribute to emotional wellness via social engagement [13].

One of the main challenge is to ensure continuous assistance in a variety of situations, adapting general capabilities of robots to the specific features of assistive scenarios. Robots should be able to act in a socially accepted manner, ensuring a user interaction as much fluid, natural and engaging as possible. Novel control approaches should thus properly deal with human needs, preferences and *uncertainty* as well as human-related interaction features like e.g., *social norms*, *culture* and the *environmental context* [4, 8]. Furthermore, emotions and empathy are crucial components of human-robot interaction [23] and can help strengthen the acceptability of technological solutions, especially in the long term. Considering frail elderly in particular, emotion is a highly relevant factor to foster their involvement during interactions.

Robots need a *mind* to effectively act in complex social scenarios and interact with humans in a reliable, continuous and adaptive way. This work describes recent advancements concerning the realization of an AI-based cognitive control approach for SAR [62, 64] exploiting Artificial Intelligence (AI) techniques to realize (artificial) cognitive capabilities of robots. Our long-term research goal is to develop a robot-integrated cognitive system enabling intelligent behaviors, engaging human-robot interactions and continuity of use during assistive tasks. The two-fold aim is, on one hand, to create a support for healthcare professionals in their role of caregivers, on the other hand, to provide assistive robots with *personalization* and *adaptability* to effectively help patients with heterogeneous health-related needs in different assistive contexts. The idea presented here is influenced by existing cognitive architectures [35, 36, 38] and pursues the integration of different AI technologies to realize the *cognitive capabilities* necessary to pursue adaptation and personalization as well as natural and engaging interactions [41, 49].

Similarly to other works [3, 51, 52], the proposed cognitive architecture is inspired by the dual process theory, a psychological model according to which the human mind would follow two distinct and parallel reasoning processes. The two processes consist of an implicit, automatic, unconscious and faster process (*System 1*) and an explicit, controlled, conscious and slower process (*System 2*) [32, 34, 46, 50, 69]. Based on this theory, we propose the Miriam architecture (where Miriam stands for “Mind Inspired aRchItecture for Adaptive huMan-robot interaction”). It integrates two interconnected “autonomous” layers working at different time scales and making decisions over different *horizons*. A *“faster"* reactive layer encapsulates *machine learning* and *natural language processing* to endow the robot with the capabilities of handling dynamic conversations and complex interactions with users in a flexible and adaptive way. A *“slower"* deliberative layer encapsulates *automated planning and execution* to synthesise contextualized assistive and supporting actions (i.e., an *assistive plan*), tailored to participants’ behaviours, characteristics, health-related needs and preferences. Additionally, this work considers the emotional-cognitive aspects of a user to support an engaging behavior and involvement of the robot. In particular, the robotic system interacts with the user through verbal and non-verbal communication channels (facial expressions) to favor an empathetic and emotional response. Miriam is evaluated on a *cognitive training* scenario where a user should follow a cognitive rehabilitation program “guided" by an assistive robot that generates a set of stimuli (or instructions) suited to the specific health needs of each user.

The article is organised as follows: Sect. 2 discusses some works on personalization and provides a brief overview of the main cognitive architectures; Sect. 3 describes the idea that inspired our architecture by describing the various AI modules on which it is based; Sect. 4 introduces the reasoning and planning module inspired by *System 2*; Sect. 5 describes the reactive module used to dynamically customise the robotic agent’s behaviour during interaction with the user inspired by *System 1*; Sect. 6 demonstrates the functioning of the proposed architecture on an example in the domain of cognitive rehabilitation and presents preliminary assessments of the system. Finally, Sect. 7 concludes the work and points to future developments.

## Related Works from Human-Robot-Interaction

### Personalization

One of the main challenges of human-robot interaction applied to different research areas such as physical and cognitive rehabilitation, support for social interaction or robot companions, lies in ensuring a fluid and effective interaction so that the user feels like interacting with an intelligent agent. Interactions based on non adaptive behaviors can become repetitive over time, decreasing user involvement after the novelty effect wears off. In this perspective, both reactivity and personalization are valid features to improve user involvement in long-term interactions, allowing an artificial agent to adopt behaviors that adapt to various relevant factors such as, for example, user’s personality, needs, preferences and the changing context of the interaction. Human-Robot Interaction (HRI) studies have been inspired by many theories of Social Psychology and behavioral research to create systems customised to the users, the context, the environment and the tasks and also to be able to react to dynamics events in long-term interactions.

The similarity-attraction principle [22], for example, assumes that individuals are more attracted to others who manifest the same characteristics. Indeed, it has been suggested that interpersonal similarity and attraction are multidimensional constructs in which people are attracted to people similar to themselves in demographics, physical appearance, attitudes, interpersonal style, social and cultural background, personality, preferred interests and activities, communication and social skills [39].

Some of the aforementioned constructs have been investigated in HRI and used to suggest best practices and guidelines to system developers. For example, social and cultural background have been explored in [8]. The authors conceived a culture-based ontology to foster contextualised HRI. A preliminary evaluation of the framework with Italian and German volunteers showed that using the framework with the proposed algorithms can significantly speed-up the acquisition of person-specific knowledge. It has also been argued that cultural robotics should design robots to be sensitive and adaptable to salient cultural factors, rather than designing robots for specific cultures, because of dynamic nature of culture itself and its role in shaping human cognition and social interaction which are equally dynamic [66]. Also *personality* has been investigated in HRI, e.g. [42, 58] to guide/influence the interaction between humans and robots. Correlations among personality traits such as sociability, activity, impulsiveness, liveliness and excitability influence implemented robot behaviors. In this regard the extroversion-introversion dimension represents one of the most used trait, possibly because of its ease of implementation in terms of behavioral expressions. Research has given attention also to both verbal and non-verbal cues. There is evidence indeed that non-verbal cues could attribute positive or negative valence to a “neutral" verbal message, and that verbal and nonverbal communication can facilitate the understanding of messages provided by the robot, when combined appropriately [33].

This work focuses on some of the mentioned aspects in order to support personalised dialogues in the cognitive rehabilitation domain where a robot is supposed to deliver tailored cognitive training to older adults.

### Cognitive Architectures

Artificial Intelligence, Cognitive Sciences, Neuroscience and Robotics have all contributed to the understanding of minds by focusing at different levels of abstraction. While Cognitive Sciences mostly focus on understanding cognitive processes and Neuroscience focus on the structure and physiology of the brain (i.e., the physiological and physical correlates of mental processes), Robotics and AI focus on understanding how minds work in order to provide software or physical agents with intelligent behaviors. Although with different perspectives and levels of abstraction, the common objective is to understand and simulate the functioning of *human mind* (or *the brain* if looking at the “physical device") and the related cognitive functions. Cognitive architectures represent those part of AI research, with the goal of creating programs capable to reason about problems across different domains, develop insights, adapt to new situations and reflect on themselves. Similarly, research in cognitive architectures aims at modeling the human mind, eventually enabling to build human-inspired artificial intelligence.

During the last years, different efforts explored the development of cognitive architectures based on the human mind (see [35] for a review). A common agreement among AI researchers sees cognitive architectures classified in *symbolic*, *connectionist*, or *hybrid* ones. *Symbolic architectures* use production rules [43] and represent concepts using symbols that can be manipulated using a predefined instruction set. In *connectionist architectures*, (e.g., [26, 44]), the adaptability and learning aspects is resolved by building massively parallel models and concepts are represented across multiple components or nodes that are organized in networks. *Hybrid architectures* attempt to combine elements of both symbolic and connectionist approaches, by including both symbolic and subsymbolic components [7], with the aim to match human cognition.

Within the hybrid architectures category some research efforts have been inspired by the dual process theory and combined symbolic and subsymbolic components in the attempt to simulate the two processes (System 1 and System 2). More specifically, [3], propose to endow a social robot with a computational explanation module based on two components: a System 1 module (S1) responsible for the fast categorization and for the perceptual based recognition of gestures in a social context, based on deep neural network architecture; a System 2 component (S2) responsible for providing a high level model that can be exploited to extract an explanation about the high level features that characterize the categorized output provided by S1 exploiting an ontology.

Differently, [51] developed a model able to handle both symbolic and subsymbolic reasoning, by means of an architechture based on two memory systems: (i) a long-term memory, an autonomous system that develops automatically through interactions with the environment, and (ii) a working memory, a memory system used to define the notion of (resource-bounded) computation. The long-term memory is modeled as a transparent neural network that develops autonomously by interacting with the environment, while the working memory is modeled as a buffer containing nodes of the long-term memory.

Finally, the Clarion architecture is a hybrid cognitive architecture with both connectionist and symbolic representations, that combines implicit and explicit psychological processes, and integrates cognition (in the narrow sense) and other psychological processes. Overall, Clarion is a modularly structured cognitive architecture consisting of a number of distinct subsystems, with a dual representational structure in each subsystem [52].

In line with these previous studies, our current work evolves from a previous effort [62] where a cognitive approach was developed to integrate contextual reasoning and goal recognition with automated planning and execution in order to realize personalized continuous and proactive assistance in daily living scenarios. While the cognitive system realized in this previous work can be classified as a *symbolic architecture*, the current work moves towards a *hybrid architecture* integrating *learning* and *runtime adaptation* capabilities and takes inspiration from the dual process theory.

### Empathetic Interaction between Humans and Robots

A further aspect addressed in current research concerns the management of emotions. Indeed, emotions are included among the crucial components in interaction and, as a consequence, they represent a key aspect also for socially interactive robots [23]. Social robots can be used to encourage emotional expression in situations where such expression may be challenging. Robots are, for instance, used to encourage children with autism to open up emotionally [21] and empathy is considered as relevant in providing assistance to older people [19, 55]. With older adults, emotion constitutes a highly relevant human factor to consider for improving user engagement while interacting with assistive robots. On one hand, psychologists showed that empathy plays a key role for therapeutic improvement (see, e.g., [48]): patients who have received empathy from their therapist recovered faster and the same seems to hold with assistive robots.

Robots can be designed to show empathy to improve users satisfaction and motivation to get better as well as enhance adherence to therapy programs in the context of patient-therapist interaction [55]. The work in [65] presents a prototype for an emoting robot that can detect emotions in one modality, specifically in the content of speech, and then express them in another modality, specifically through gestures. The robot is able to detect and express emotions through an emoting system. Results from a human validation study shows people perceiving the robot gestures as expressing the emotions in the speech content. Also, people’s perceptions of the accuracy of emotion expression is significantly effective. In [15], an affective reasoning system has been implemented in the NAO robot for simulating empathic behaviors in the context of AAL. In particular, the robot recognizes the emotion of the user by analyzing communicative signals extracted from speech and facial expressions. The recognized emotion allows triggering the robot’s affective state and, consequently, the most appropriate empathic behavior. Such behaviors have been evaluated both by experts in communication and through a user study aimed at assessing the perception and interpretation of empathy by elderly users. The above work considers emotions, empathy, feelings and affections as relevant features in human-to-human interactions.

In line with this research, the objective of this work is to design social robots capable also of detecting and expressing emotions and showing empathy so as to deploy empathetic interaction dialogue and foster engaging communication.

## MIRIAM: a Mind-Inspired Architecture for Adaptive HRI

The technological contribution of this work is inspired by the psychological theory of dual process. From its initial formulation by William James [32], several proposals and interpretations have been made through years. Peter Wason and Jonathan Evans suggested a dual process theory in 1974 [69] by identifying two distinct types of processes: heuristic processes and analytic processes. Richard E. Petty and John Cacioppo proposed an application focused in the field of social psychology in 1986 [46], known as *the elaboration likelihood model of persuasion*, and more recently, Daniel Kahneman provided further interpretation by differentiating the two styles of processing more, calling them intuition and reasoning in [34]. Similarly, Strack and Deutsch [50] identified two separate systems: the reflective system and the impulsive system. Despite minor differences due to its evolution, there is a common agreement that the dual process theory accounts on two systems for explaining how thoughts arise from different processes which consist of an implicit, automatic, unconscious process (System 1) and an explicit, controlled, conscious one (System 2).

Without pretending to match cortical structures of the brain into software modules, we propose Miriam as a novel AI-based control architecture that bring to different (but intertwined) thinking styles: the System 1, implicit and automatic, is mirrored into a faster and reactive layer; the System 2, explicit and controlled, is mirrored into a slower and deliberative layer.

### The Miriam Architecture

AI technologies like *Automated Planning* (AP), *Knowledge Representation & Reasoning* (KR &R) and *Reinforcement Learning* (RL) support *reasoning capabilities* that, to some extent, replicate cognitive functions typical of System 1 and System 2. These technologies, taken alone, would not support higher levels of behavior adaptation and flexible control. A proper integration of these technologies would be crucial to enhance the efficacy of robots in scenarios that entail a tight and continuous interaction with humans.

AP [31, 47] is well suited to synthesize a complex set of actions supporting the desired assistive objectives but, it lacks of the flexibility and adaptability needed to naturally interact with humans. KR &R [28, 37, 59] is well suited to represent the domain features of assistive scenarios and support contextualized reasoning but, it lacks of a “runtime perspective" and is affected by the same limitations of AP. General rule-based AI systems tackle problems with a high level of abstraction, generating solutions that maintain a general and long-term view of a problem. However, they struggle in generating solutions that are flexible enough to effectively face unpredictable and unforeseen changes that are typical of human behaviors. On a different side RL [53] has the reactivity levels and the adaptation capabilities desired to deal with human dynamics. It is indeed quite effective in environments that change dynamically and unpredictably. It incrementally builds/refines an internal model (or *policy*) from observations through *rewards* in order to dynamically adapt choices and executed actions to different *situations*. One of the limitations of RL however is a lack of a “long-term perspective" as well as the “semantics" and the “explicit structures" needed to properly explain behaviors and failures [17].

**Fig. 1:**
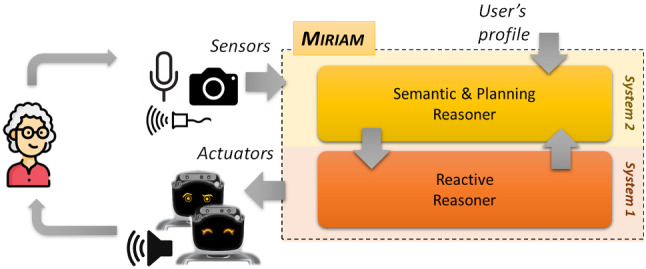
A sketch of the architecture

Given these premises, it is not hard to map RL to Systems 1 and, AP and KR &R to Systems 2. Figure 1 shows the resulting structure of Miriam which consists of two layers, each associated to a specific cognitive process (i.e., thinking style). The architecture aims at combining the benefits of the mentioned AI technologies and thus achieve abstract and goal-oriented reasoning while maintaining high reactivity and adaptation. Focusing on dialogue-based interactions with humans, Miriam would dynamically deal with contingent behaviors perceived through robot sensors while producing personalized behaviors through its adapters like e.g., sentences pronounced in natural language or appropriate facial expressions. The dynamic modelling capabilities of the system would for example adapt the communication modality of the robot (e.g., verbal and non verbal) to known *personality insights* as well as changing mood of a user. As it will be better described later in Sect. 6, taking inspiration from [56], the extroversion-introversion trait of the Big Five Personality model is taken into account for modulating the robot’s behavior. The robot will thus use reassuring words and eyes expressions in case of *introverted users* while, it will use challenging words and eyes expressions in case of *extroverted users*. The combination of the two reasoning layers thus allows a robot to achieve (long term) objectives by carrying out (abstract and detailed) plans in a personalized and adaptive way, based on to the features of users.

From a technical perspective we design the interactions between System 1 and System 2 layers by “revisiting" Hierarchical Reinforcement Learning (HRL) [54] where a upper-level policy (often referred as the *gating policy*) learns to select and communicate *motivations* (or *options*) to a lower-level policy (often referred as *option policy*) which learns concrete behavior/control patterns. Unlike HRL, however, we replace the gating policy with AP and KR &R technologies in order to carry out complex reasoning tasks, while maintaining the reactivity characteristics of the option policy. Following this nomenclature we refer to higher level actions planned (and executed) by System 2 as *motivations* and refer to lower level physical actions (or interactions) performed by System 1 as *actions*. Sects. 4 and 5 show with more details how the two layers of Miriam are internally structured and how AI technologies support the related reasoning and interacting capabilities. Although the paper specifically focus on dialogue-based interactions between a human and a robot, it is important to point out that Miriam can support different types of interactions. The layer of System 1 can indeed be easily extended with additional “reactive modules" supporting different interaction skills of a robot (e.g., object manipulation skills or navigation skills). The reasoning capabilities of System 2 would seamlessly interact (and coordinate) the reacting modules composing the System 1 maintaining the same level of abstraction.

## Semantic Reasoning and Planning

The reasoning capabilities realized at System 2 level rely on the tight integration of KR &R and AP. This layer specifically integrates the ontology-based and contextual reasoning capabilities of KOaLa (*Knowledge-based cOntinuous Loop*) which proposes a *holistic* approach to robotic assistance [64].

KOaLa supports the definition of a context-based *knowledge* that endows a robot with a semantically rich representation of assistive scenarios. Contexts specify robot knowledge at different levels of abstraction and enable *contextual reasoning* to process raw sensory data and/or input health information about users. For example, as shown in [62], KOaLa integrates the *Semantic Sensor Network* (SSN) ontology [12] to interpret data gathered from environmental sensors (e.g., PIR or SWITCH sensors) and *recognize* daily activities of a user at home. Furthermore, KOaLa integrates an ontological representation of the *International Classification of Functioning Disability and Health* (ICF) proposed by the World Health Organization^1^ in order to reason about the *health conditions* of users and contextualize assistance accordingly. Leveraging the foundational ontology DOLCE [24], elements of ICF are represented as *functioning qualities* (i.e., specializations of DOLCE:Quality) characterizing functioning levels of physical and/or cognitive aspects of a person.

In [62] it has been shown that the combination of context-based KR &R with AP was effective in synthesizing proactive assistance. Plans and assistive actions of a robot were indeed dynamically synthesized according to the *goals* automatically triggered from inferred daily-living situations. The current work further extends KOaLa by refining the underlying ontological model and the integration with planning.

### Deploying KOaLa into Miriam

To support a wider range of assistive scenarios the ontological model has been extended to formally characterize the *assistive capabilities* of a robot. Robot skills are interpreted as physical or cognitive *stimuli* with respect to the *health state* of a user. The ICF classification defines a reference framework to formally characterize the *effects* that assistive actions (i.e., stimulus) have on the (ICF-based) health variables describing the state of a user. As shown in [63], this allows the reasoning layer to contextualize assistive capabilities of a robot and dynamically identify the (sub)set of capabilities (and thus stimuli) that best fit the specific health-related needs of the assisted user.

Robot capabilities and related stimulation actions are defined according to the (positive) *effects* they have on the functioning qualities of a person. In the case of cognitive stimulation, for example, stimulation actions consist in administrating some *known* cognitive exercises to a user through some interaction modality. The capabilities of such actions (i.e., the *effects* they have on the health state of a person) depend on the capabilities of administrated exercises (i.e., the functioning qualities targeted by the exercises).

**Fig. 2:**
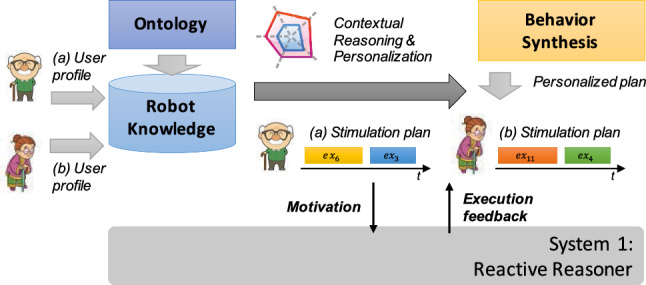
Internal components of System 2 level

This ontological model combined with input *user profiles* constitute the internal *knowledge* of a robot at System 2 level. This knowledge, together with the reasoning modules that dynamically identifies suitable actions and synthesize personalized assistive behaviors, compose the System 2 layer of Miriam architecture as shown in Fig. 2.

A *user profile* instantiates the ICF-based ontological model and describes the health state of a specific person through a set of *numerical scores* describing the functioning levels of “measured" physical and/or cognitive qualities. Such scores are defined according to ICF (i.e., 0 - no impairment; 1 - soft impairment; 2 - medium impairment; 3 - serious impairment; 4 - full impairment) and represent the level of *impairment* of a modeled functioning quality like *memory functions* (e.g., *short-term memory*) or *attention functions* (e.g., *sustaining attention*). A user profile is internally represented as a *Knowledge Graph* associating to each *functioning quality* of the ontological model one of the described ICF-scores. Namely, a profile consists of a number of *measurements* each associating an ICF-score to a specific functioning quality of the ontological model. In this way, a profile characterizes the cognitive and physical functioning of a user.

Knowledge reasoning mechanisms carry out *contextual reasoning* to *infer* physical and/or cognitive *impairments* of a user and accordingly contextualize robotic assistance. Such reasoning mechanisms are implemented through a custom rule-based reasoning engine developed using the open-source software library Apache Jena^2^. Equation 1 and Equation 2 show the general logical rules integrated into KOaLa to analyze health conditions of a user and contextualize robot capabilities. Equation 1 first analyzes a given *user profile* in order to recognize the (sub)set of functioning qualities that actually represent *impairments*. Impairments characterize the health conditions of a user and determine the kind of assistance a user needs. Eq. 2 then contextualizes impairments with respect to known capabilities of a robot. Robot capabilities determine the supported *stimulation functions* each of which has effects on a sub-set of functioning qualities of a user. The rule specifically infers a set of *stimulation opportunities* by taking into account the stimulation functions that have effects on the impairments of a user. As shown in [63], this rule relies on a formalized concept of *affordances* that characterize opportunities of performing stimulation (and thus assistive) actions. 
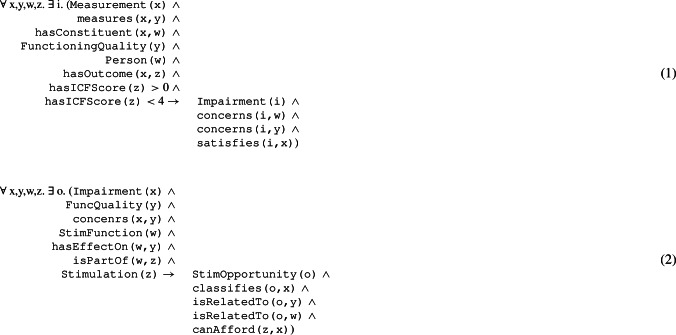


### Personalizing and “Motivating” Assistance

The reasoning mechanism implemented through KOaLa allows a robot to “understand" health conditions of a user and autonomously recognize stimulation actions that fit her needs. Contextual knowledge is given to the *behavior synthesis* component which synthesizes and executes personalized assistive plans addressing the specific needs of a user. As shown in Fig. 2 indeed users with different health needs (i.e., different impairments) lead to the synthesis of different stimulation plans and, for example, to the administration of different cognitive exercises.
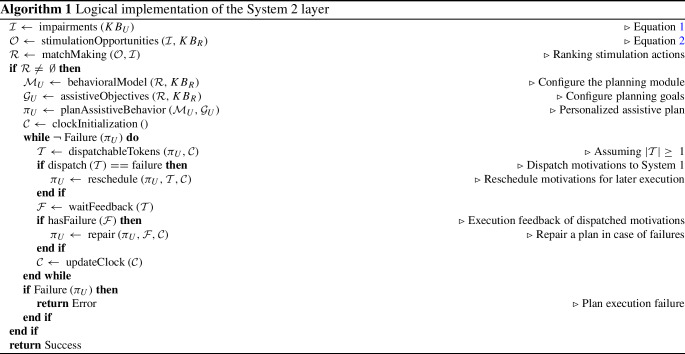


Algorithm 1 shows the functional steps that support the reasoning capabilities of System 2 and guide assistance through System 1. The set of impairments $\mathcal {I}$ and *stimulation opportunities* $\mathcal {O}$ inferred through Eq. 1 and Eq. 2 characterize the *contextual knowledge* of the robot. This knowledge is further elaborated through a *match making* mechanism aims at identifying the most suitable actions a robot can perform to support a user. A *recommended system engine* generates a *ranking* $\mathcal {R}$ of stimulation actions by correlating impairments and stimulation opportunities. In the case of cognitive stimulation for example, the ranking $\mathcal {R}$ characterizes the set of exercise administration actions that address the most impaired functioning qualities of a user. These actions are supposed to be the most effective in addressing the specific health conditions of a user (*personalization*). The obtained ranking $\mathcal {R}$ is used to dynamically configure the AP module by defining a personalized *planning model* $\mathcal {M}_U$ and a personalized set of *assistive objectives* $\mathcal {G}_U$ (i.e., planning goals). This allows the reasoning layer to synthesize a *personalized assistive plan* $\pi _U$ that takes into account the specific health-related needs of a user and the sub-set of suitable assistive capabilities of a robot.

The implemented planning and execution capabilities rely on the timeline-based formalism [11] and the open-source PLATINUm framework^3^ [60]. Assistive plans are thus represented in terms of timelines [11] that describe the sequences of (high-level) assistive actions and/or stimuli, called *tokens*, a robot should performs over time. Planned assistive actions represent *motivation signals* that are interpreted by System 1 to (physically) interact with a user. As shown in Algorithm 1, the execution of a (personalized) plan $\pi _U$ at System 2 level consists in “dispatching" a number of planned *tokens* $\mathcal {T}$ to the reactive layer of Miriam. Such tokens thus represent the motivation signals that guide the interactions carried out by System 1 to achieve high-level assistive objectives of plan $\pi _U$.

In the case of dialogue-based interactions and cognitive stimulation for example, motivations (i.e., the tokens of the timeline-based plan $\pi _U$) represent the administration of cognitive exercises and specify the type of dialogue a robot should carry out. Every time a token of a timeline is about to start according to the current clock $\mathcal {C}$ and its schedule, a properly configured *motivation* is dispatched to System 1 for the administration of a particular cognitive exercise. System 1 sends back *feedback* about dispatched motivations. Three cases can be distinguished as shown in Algorithm 1: (i) if System 1 is not ready for the execution of the dispatched motivation (*dispatching failure*) (e.g., the user refuses to start a cognitive exercise) the related token is *rescheduled* for being executed later in the plan $\pi _U$; (ii) if System 1 is not able to correctly complete the execution of dispatched motivations (*execution failure*) (e.g., the quality of the dialogue was too low or the user made too many mistakes) then a *plan repair* is performed (e.g., additional exercise administration actions are planned to achieve the desired level of “daily stimulation"); (iii) if System 1 correctly executes motivations (*positive feedback*) (e.g., the administrated exercise has been successfully completed by a user) the execution of plan $\pi _U$ proceeds with the rest of planned tokens.

It is important to point out that, the kind of (slower) contextual reasoning performed at System 2 level is here used to *personalize* both the *content* of the assistance i.e., *which* actions are suitable to achieve the desired objectives, and the *shape* of the assistance i.e., *how* actions should be executed to be effective. To support such personalization the ICF-based ontological model characterizes *interaction capabilities* of a person in order to properly set the *modality* of the interaction used by the robot. These capabilities mainly concern hearing and seeing functioning qualities of a user in order to configure parameters like e.g., the *sound level* of auditory messages or the *font size* of text messages. Other relevant aspects concern the *personality* of the assisted person and the *social context* where the assistance takes place in order to properly set the way a robot *approaches* a person, *attracts* her attention and the way the robot communicates with him/her. Dispatched tokens (i.e., *motivations*) are thus “tagged" with a number of *interaction parameters* that provide the reactive layer with contextual information about their execution [14]. Section 6 shows some examples describing the supported behaviors.

## Reactive Reasoning

**Fig. 3:**
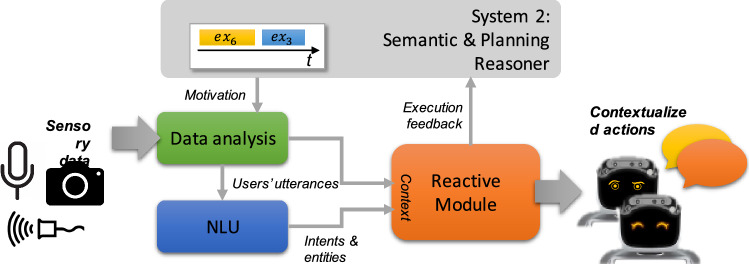
Internal components of the Reactive Reasoner (System 1)

Unlike the System 2 described in the previous section, which by carrying out higher level forms of reasoning synthesizes a sequence of motivations to be executed over time, the role of our System 1, sketched in Fig. 3, consists in selecting actions based on a context that dynamically evolves over time. In other words, the objective of the reactive module consists in building a *policy* $\pi \left( ctx \right) = a$ which, given a context *ctx*, returns an action *a* to be executed. The execution of such actions, in particular, physically translates, in our case, in the pronunciation of personalized sentences in natural language towards a user and, similarly to [10], in the contextualized change of facial expressions. Since, however, these actions internally constitute transitions in a *state-transition system* [16], we propose a new form of parametric actions, inspired by those used in classical planning [25], which allow to represent the state-transition system in an implicit and compact way. Before describing these actions, however, it is advisable to define the states of such a state-transition system which, by analogy with natural language generation systems, we will call context.

From a technical perspective, we refer by *context* to a set of variables, both symbolic and numeric, which characterize the current state of the system. These variables are used to keep track of all the information that, more or less dynamically, change over time. Specifically, context variables include all those factors which are relevant for the discrimination of the actions taken by the system such as, for example, those related to the user’s personality and current mood together with those elements related to the interaction like, for example, information extracted from the user’s speech analysis and the robot’s facial expressions (refer to Table 3 for a description of some of the context variables used by the system). By dynamically updating the values of the context variables, the system will adaptively select actions with the aim of personalizing the interaction and obtaining dialogues and, more in general, behaviors, that are as fluid and engaging as possible.

Once introduced the context, on the basis of which the policy selects the actions, we can describe in more detail the actions. Each action, in particular, is characterized by three elements: (a) a logical combination (i.e., conjunctions and/or disjunctions) of *conditions* on the context variables for verifying the executability of the action; (b) a set of natural language sentences representing the system’s *responses* for the users (if the set contains more than one sentence, one is chosen randomly); and (c) a set of *effects* on the context variables, representing the updates to apply on both symbolic and numeric context variables whenever the action is executed, as well as the possible feedback for the System 2, indicating the termination, either successful or unsuccessful, of the current motivation.

Similarly to classical planning, each action whose conditions are verified in the current context is said to be *executable*. As opposed to planning, however, whenever asked to the system, i.e., as a consequence of the interactions from a user or for the incoming of a motivation signal from the System 2 module, all executable actions are executed in the order they are defined by the domain author. The presence of such actions, indeed, is intended both to establish which responses to provide to the users and to make further transitions in the context space by means of the actions’ effects updating, for example, some context variables about the current discussion topic.

The updating of the context variables, in particular, takes place as a consequence of the transmission and interpretation of high-level commands, which we called motivation signals, coming from the execution of the customized plans. These variables, however, are also modified as a consequence of the unpredictable interactions that the system has with the users. By analyzing the data coming from the sensors (in particular, the signal from a microphone), a speech-to-text module^4^ recognizes text from users’ speech. This text is subsequently analyzed through a Natural Language Understanding (NLU) module^5^ which is trained to classify the users’ intentions (e.g., affirmations, negations, answers to questions, etc.), called *intents*, and to annotate them with further data, called *entities*, to better characterize them (e.g., numbers or dates in answers). Whenever a user utters a particular sentence, in particular, this high-level information is extracted and used to modify the values of the current context variables, triggering the selection of the proper action which, in turn, results in the proper reaction by the system.

One aspect that is worth highlighting concerns the customization of the system according to the user’s personality. In particular, any sentence pronounced by the user, once recognized through speech-to-text, is sent to the NLU module for the recognition of intents and entities and, at the same time, is stored. Through a personality insight tool, starting from the set of all the sentences uttered by the user, we extract information on the personality such as extroversion, neuroticism, etc. These parameters, once again, enrich the set of context variables, further personalizing the interaction with the user by implementing, for example, a more challenging type of communication for an extroverted person or a more accommodating type of communication for a more introverted person.

Algorithm 2 shows, in pseudo-code, the steps that are performed every time the robot is requested to interact with the user (i.e., when a motivation signal is received by the deliberative layer or when the user says something). In particular, for each action whose conditions are satisfied in the current context, a response is randomly selected and proposed to the user and, subsequently, the context variables are updated according to the effects of the action. Once all the actions have been processed and the context updated with the effects of the executable ones, the algorithm first checks the state of a particular context variable (i.e., *eyes*) indicating the eyes to show and, depending on its value, modifies the robot’s facial expression to make the interaction more empathetic. Finally, it checks the status of a second context variable (i.e., $command\_state$) indicating the status of the high-level command triggered by the deliberative layer and, if it takes on specific values (i.e., *done* or *failure*), it communicates at the deliberative level (respectively, with a success or a failure) the conclusion of the task.
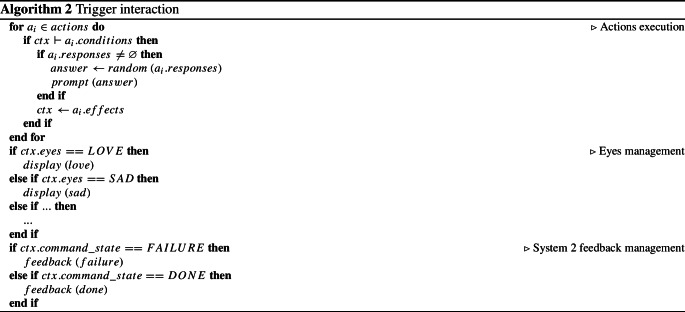


It is worth specifying that among the parameters extracted from the speech-to-text module and from the NLU module, a confidence value indicates how confident the speech-to-text module is in recognizing a sentence from the audio signal and how confident the NLU module is in classifying the user’s utterances. This same value is added to the different context variables, further adapting the interaction. In particular, if the confidence value falls below a certain threshold, the robot can ask the user to rephrase the last sentence differently or, in the event of repeated misunderstandings, the actions performed by the reactive module could bring the status of the current command to a failure and then to a feedback for the deliberative module. Finally, note that just as the eyes are managed, the reactive module can easily be extended to drive a richer multimodal interaction, controlling gestures and modulating prosody.

## Applying MIRIAM

The target scenario foresees a training service where the robot is used for delivering a cognitive rehabilitation program. An assistive robot should generate a set of stimuli (or instructions) suited to the specific health needs of a user (i.e., the assisted person) and administrate these stimuli (e.g., cognitive exercises) through a dynamic, engaging and natural interaction.

### From User Profiles to Personalized Plans

To assess the reasoning (and personalization) capabilities developed at System 2 level of Miriam, we have made a test on 8 (real) user profiles that are characterized by different cognitive states. These profiles were obtained after a clinical assessment made by a clinician using a standard cognitive evaluation tool, i.e., the Mini Mental State Examination (MMSE). Table 1 succinctly shows the obtained profiles that are internally represented as *knowledge graphs*. For each profile the table shows the considered ICF variables and the associated numeric scores (0 – no impairments; 1 – soft impairment; 2 – medium impairment; 3 – serious impairment; 4 – full impairment).

**Table 1:** User profiles and associated ICF variables

ID	ORI	ATT	MEM	PER	HLC	MFL	CAL	COM	SPK	WRT
1	2	4	3	2	2	0	4	1	0	0
2	2	3	3	3	3	3	3	2	2	4
3	1	0	0	3	0	1	0	0	0	4
4	3	2	3	4	3	1	2	2	0	4
5	1	0	0	0	0	1	0	0	0	4
6	1	4	3	3	2	0	4	0	0	0
7	3	2	3	3	2	2	2	0	2	3
8	1	4	3	4	2	2	4	0	2	3

*ORI* Orientation; *ATT* Attention; *MEM* Memory; *PER* Perceptual; *HLC* Higher Level Cognitive Functioning; *MFL* Mental Functioning of Language; *CAL* Calculation; *COM* Communication; *SPK* Speaking; *WRT* Writing

Furthermore, the knowledge of the robot was filled with a total number of 10 cognitive exercises each addressing different (cognitive) aspects of a user. Each exercises was internally associated to a distinct stimulation action (i.e., exercise administration action) and thus modeled as a distinct cognitive capability of the robot (i.e., stimulus). Table 2 shows the considered exercises the associated stimulation functions and thus the list of stimulated variables of the ICF-based profile of a user.

**Table 2:** Known cognitive exercises modeled as stimulation capabilities

ID	Exercise Name	Stimulated ICF Variables
1	Denomination test	MFL
2	Find the word	MEM, CAL
3	Free and cued selective reminding test	MEM
4	Stroop test	ORI
5	Animal test	HLC, MFL
6	Backward digit span test	MEM, CAL
7	Reys’s figure test	MEM
8	Trailing making test form B	ATT, HLC
9	Trailing making test form A	ATT, HLC
10	Boston naming test 40-items	MFL

**Fig. 4:**
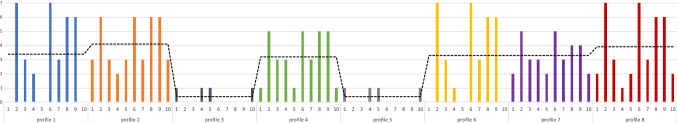
Ranking of stimulation capabilities for the considered user profiles. The dotted line shows for each profile the computed average ranking values of the exercises

Following the steps sketched in Algorithm 1 the reasoning layer analyzes the *knowledge* inferred from the information contained in Table 1 and Table 2 to first contextualize stimulation capabilities and then synthesize (and execute) personalized plans. Rankings are computed by multiplying vectors of ICF scores assigned to each profile (i.e., rows of Table 1) with a “incidence matrix" extracted from stimulation capabilities of cognitive exercises. For each exercise (i.e., *simulus*) the matrix would contain a positive value (i.e., 1 in the current implementation) if a particular ICF variable is supported/stimulated or 0 otherwise. This reasoning process implements a *knowledge-aware recommender system* [2, 29] leveraging the underlying (ICF-based) ontological model to contextualize health-related needs of patients with stimulation capabilities of known stimuli (i.e., *assistive affordances* [63]). A qualitative view of outcome of the ranking procedure aiming at characterizing the relevance of the modeled stimulation capabilities with respect to the health (cognitive) conditions of different users can be seen in Fig. 4.

**Fig. 5:**
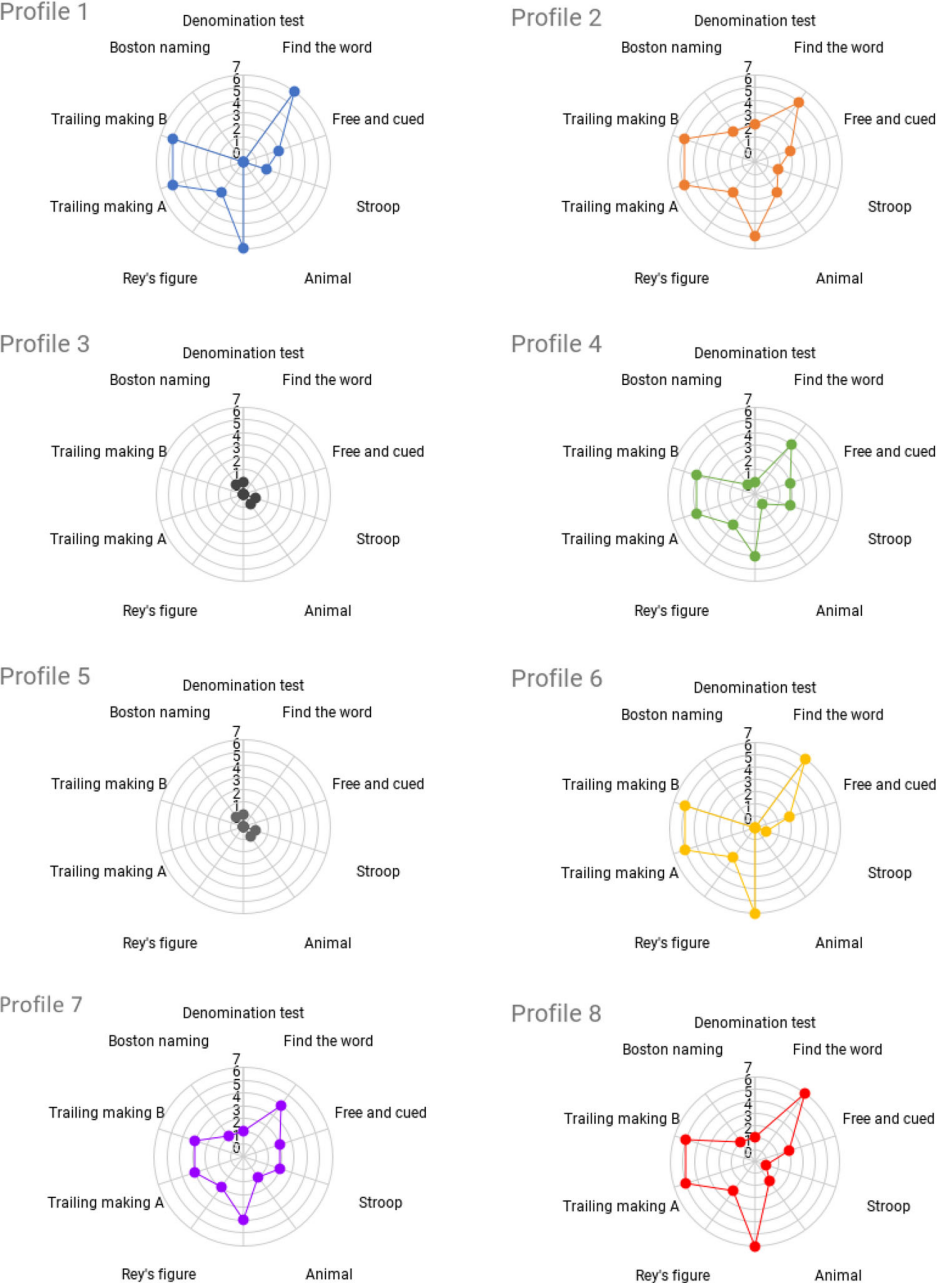
Detailed view of rankings with respect to the stimuli of Table 2

At a first glance it can be easily seen how the reasoning layer is actually able to differentiate the *intervention* for the considered profiles. Different *ranking values* (i.e., relevance) were computed according to the health state of each user (Table 1) and to the specific stimulation capabilities of each exercise (Table 2). For example, low ranking values were computed for users with no significant impairments like e.g., *Profile 3* and *Profile 5*. Conversely, several exercises were detected as *relevant* i.e., “high^6^ ranking value" for users with more serious conditions. An example is *Profile 8* which is characterized by medium and serious impairments (respectively {ATT, PER} and {MEM, WRT}). In this case different exercises achieve a not negligible ranking value. However, the exercises that are considered as *relevant* are *Exercise 2, Exercise 6*, *Exercise 8* and *Exercise 9* whose ranking value is higher than the average. Similar results can be seen for *Profile 1*, *Profile 6* or *Profile 2*. A closer view to the results is available in Fig. 5 showing rankings obtained for each user profile and clearly pointing out the correlations with the modeled stimuli of Table 2.

The obtained results seem coherent with respect to the health conditions described by the considered user profiles. The contextual knowledge has been thus used to configure the timeline-based planning and execution model used to actually synthesize and execute such (high-level) personalized stimulation plans. In this specific case, the planning model has been dynamically synthesized by taking into account, for each profile, all and only the stimulation actions that were inferred as relevant. In this way, synthesized (timeline-based) plans contains only stimulation actions that are suitable for the specific health state of the assisted person. System 2 thus will dispatch to System 1 *motivations* requesting the administration of cognitive exercises that actually stimulate impaired (cognitive) qualities of a user.

Listing 1 below shows an excerpt of a timeline-based plan $\pi _U$ synthesized to administrate a number of cognitive exercises (8) for a particular patient. Three timelines compose the plan: (i) a *Stimulation timeline* describing the high-level assistive goals achieved by the plan; (ii) a *Patient timeline* describing known interaction preferences over time and; (iii) a *Robot timeline* describing the planned (and scheduled) stimulation actions of the robot (i.e., motivations dispatched to System 1 architectural level). It is worth noticing that the *Patient timeline* encapsulates preferences extracted from a user profile determining the way the robot interacts with her/him to administrate cognitive exercises. Namely, this timeline describes *when* a patient is expected to be available to receive stimulation (i.e., tokens with predicate *Available*) and *how* (the parameters of the predicate *Available* characterize *interaction preferences* of a patient like e.g., volume, need for subtitles or need for explanations). Tokens of *Robot timeline* are planned according to these preferences. Tokens with predicate *DoStimulationAction* are indeed scheduled during expected availability windows of the patient (i.e., tokens with predicate *Available*) and “grounded" according to associated interaction parameters. Temporal constraints of the plan are represented as *relations* between (flexible) temporal intervals of the tokens of the plan [1]. The Listing 1 shows just few of them (i.e., some *during relations* constraining robot stimulation tokens to be scheduled within the same temporal interval of patient availability tokens).
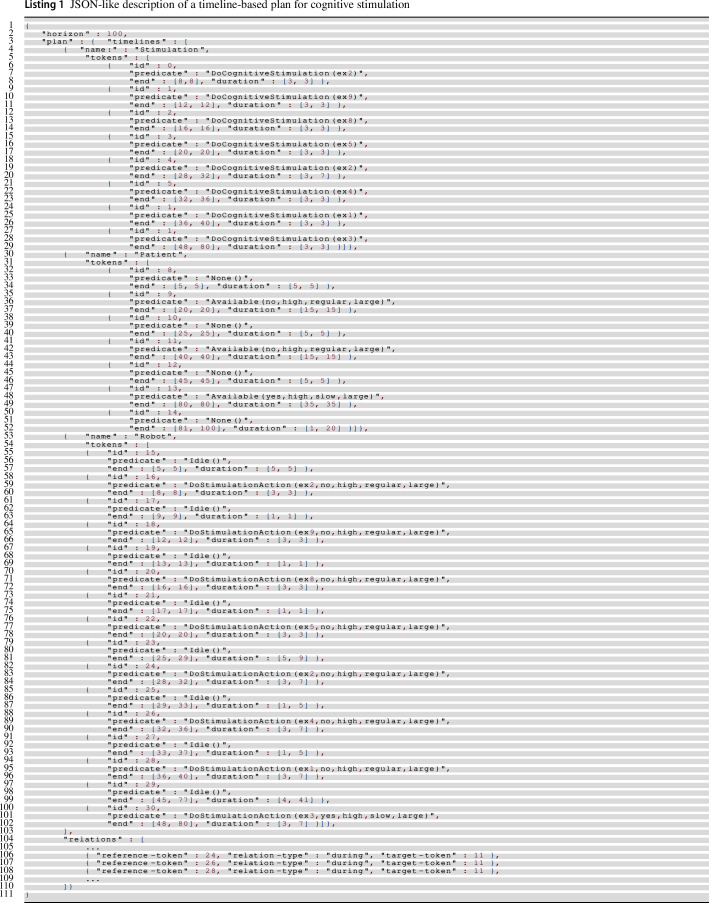


**Fig. 6:**
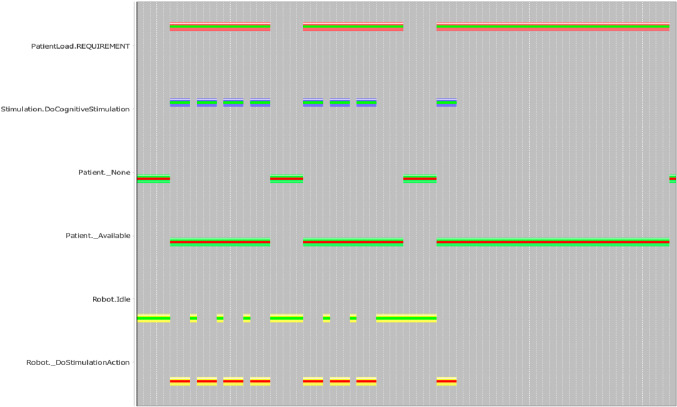
Gantt representation of an executed timeline-based plan

Figure 6 shows then a (simplified) Gantt representation of the executed plan. In this case tokens are not flexible but rather scheduled at specific (non-flexible) temporal intervals. Namely each token has start and end time points rather than intervals. The actual duration of each token is determined according to the *feedback* received from System 1 after the actual execution of dispatched motivations. Furthermore, the Gantt shows the *PatientLoad* discrete resource [61] (not showed in Listing 1) that was used to limit the maximum number of exercises administrated during a single interaction window (i.e., a particular availability token).

### Adaptive Dialogue Execution and Interaction Assessment

**Fig. 7:**
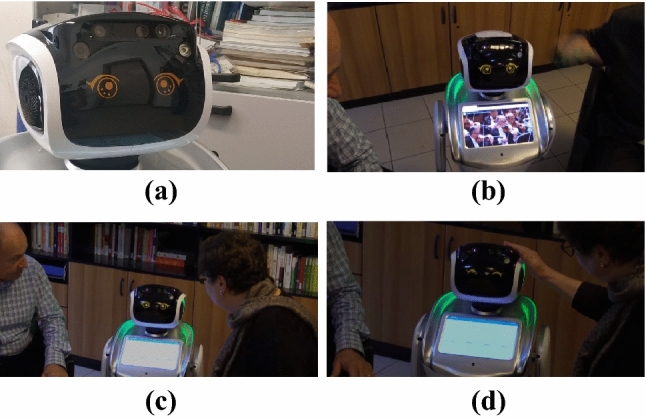
Deployment of Miriam on a Sanbot Elf

Figures 7 shows the social robot on which Miriam has been developed. The robot is a Sanbot Elf, distributed in Italy by Omitech^7^, and the figures show a preliminary laboratory deployment for testing and feasibility assessment. From the hardware perspective, the robot has a tablet, which can be used to interact with people, as well as “eyes” (see Fig. 1 and Fig. 7) that can be controlled to represent Miriam’s facial expressions (e.g., happiness, amazement, sadness, etc.). The robot has also actuators to navigate around a room and moves its head and arms. As for the sensors, the robot has two microphones used in Miriam to recognize the speech of users and several contact sensors along the whole body, several proximity sensors at the base of the robot and two cameras (one of which is a depth camera). The software architecture of the Sanbot Elf relies on a core ROS layer supporting a direct interface to the sensors/actuators that compose the robotic platform. On top of this layer Android API provides developers with a modular and programmable interface to support the integration (and interaction) of complex Java-based application and services. Therefore, the main modules developed to support the reasoning functionalities of Miriam are developed in Java which guarantees modularity, platform independence and flexible deployment on other robots^8^.

As a feasibility assessment, let us suppose that the assistive robot is capable of administrating a set of cognitive exercises $E = \{e_0, e_1, e_2, e_3, e_4, e_5, e_6, e_7\}$ and that a subset $E_u = \{e_3, e_4, e_5\}$ of these exercises has been inferred as relevant for a user *u* by the reasoning layer, according to the specific cognitive needs identified. Additional parameters managed by the reasoner layer to support the user personalization are for example, the preferred sound level of robot audio messages, the preferred speech-rate, the preferred font size of robot text messages, and possible information about social norms. Once the reasoning layer has planned the proper cognitive exercise for the target user, the reactive module is in charge of executing the plan by initiating the interaction and properly react to the dynamism of the occurring dialogue.

**Fig. 8:**
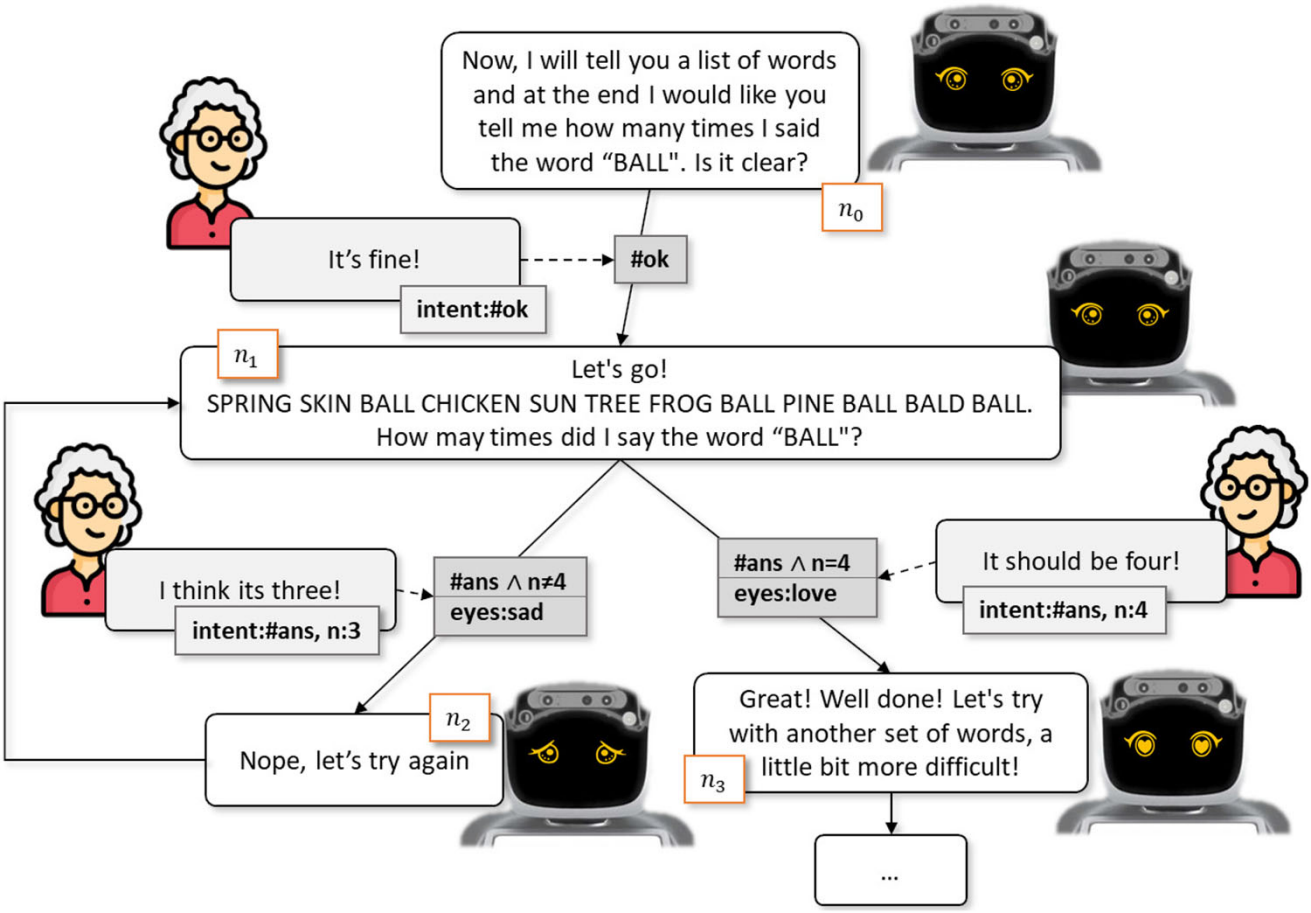
A personalized dialogue sketch showing adaptation to different users’ responses

**Fig. 9:**
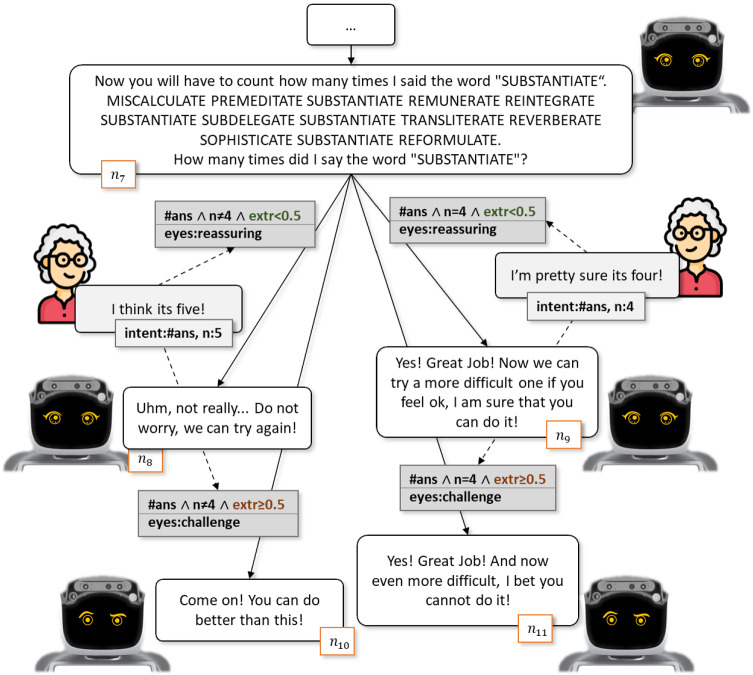
A personalized dialogue sketch showing possible answers based on users’ personality

Figures 8 and Fig. 9 exemplify the reactive behavior of Miriam while administering the *Find the word* exercise. This exercise requires the user to count the occurrences of a target word while the robot lists some predefined words, varying the difficulty according to the complexity of the used words: easy words, complex words and non-words. Beside the information provided by the System 2 which allows to tailor the interaction since its very beginning, it is showed the capability of the robot to dynamically adapt during the interaction according to contingent information and react in a multimodal manner: beside verbal channel, the robot communicate also through a visual one, by displaying gaze according to the specific message it is supposed to convey.

**Table 3:** Some of the main context variables with their initial value and a brief description

Name	Init value	Description
intent	*none*	Used for representing the user’s intents. Values are set by the NLU module
n	*none*	A numeric variable used for recognizing correct/incorrect answers. Values are set by the NLU module
sentiment	0	A numeric variable ranging from –1 to 1 representing the sentiment, from negative to positive, of the last utterance from the user. Values are set by the the NLU module
extraversion	*none*	A numeric variable ranging from 0 to 1 representing the extraversion of a user. Values are set by the personality insight module
confidence	*none*	A numeric variable ranging from 0 to 1 representing the confidence of the system in recognizing a user’s utterance or the user’s intent. Values are set by the speech-to-text module and by the NLU module
eyes	normal	Used for representing the current state of the eyes. Values are set by executing actions
node	*none*	Used for representing the current state-transition node of the exercise. Values are set by executing actions
num_errors	0	Used for representing the current performance of the user in terms of the number of errors made. Values are set by executing actions
audio_volume	normal	Used for adapting the audio volume for persons with hearing impairments. Values are set by KOaLa through motivation
exercise	*none*	Used for describing the current rehabilitation excercise. Values are set by KOaLa through motivation

The exercise, in particular, is initiated by the System 2 through the execution of the personalized rehabilitation plan and, more in detail, by sending a motivation signal to the System 1. This signal translates into the assignment of some of the context variables shown in Table 3 which denote, for example, the moving to node $n_0$ of the exercise, as shown in Fig. 8, and that, given the person’s auditory characteristics, the exercise must be reproduced at a normal volume. At the same time, the robot initiates the dialogue by providing the user with the instructions for the cognitive exercise.

At this stage, the answer from the user (let us suppose that it is a sentence like “It’s fine!”) is recorded through a microphone and translated into text through the speech-to-text service. The recognized text is subsequently analyzed by the NLU service which extracts the intent $\#ok$ updating the “intent” context variable. An action, whose execution condition is being in node $n_0$ *and* the “intent” context variable assuming the $\#ok$ value, is selected and, as a consequence of its execution, the robot starts listing the words and ask for the users to answer (in the responses of the action) and the transition to $n_1$ node (in the effects of the action). Finally, if the users provides the wrong answer, then the robot selects the proper action, increasing by one a counter of the errors made by the user, displaying sad eyes to the user and inviting him to retry the exercise ($n_{2}$). On the contrary, if the answer is correct, the robot selects happy eyes and congrats with the user ($n_{3}$).

Note that the nodes represented in these figures are only used for explanatory reasons, not explicitly representing states of a state-transition system which, on the contrary, are identified by the combination of the values assumed by the different context variables. The introduction of actions, indeed, as described in Sect. 5, allows an implicit definition of the state-transition system, facilitating the definition of the domain and allowing complex behaviors that would be difficult to model explicitly such as, for example, users asking questions out of the scope of the exercise *while* they are conducting it, without losing the focus on the exercise. Additionally, it is worth underscoring that the planner could not predict the user’s response and, therefore, could not include it in the initial plan. In the absence of the reactive module, in particular, the reaction to the user’s response would have required a potentially expensive adaptation of the plan.

Let suppose that the dialogue is going on and the robot keeps interacting with the user and dynamically acquiring variables from the context. Among these variables, shown in Table 3, also Personality is considered and inferred by the Insight tool. The system memorizes and analyzes the different interactions with the user over time and, by means of personality insight tools can, for example, collect information on the *user model* through aspects such as the Big Five personality traits (i.e., openness to experience, conscientiousness, extraversion, agreeableness and neuroticism), and on the current mood, such as a form of sentiment analysis. This information, in particular, enriches the context by updating the corresponding variables and hence further fosters the system’s personalization.

Indeed, Fig. 9 shows that the system, while interacting with the user, starts to further personalize its interaction. Different ways to react by the users brings the robot to interact in different manner according, for example, to different levels of extraversion. In particular, the system can respond more reassuringly both verbally and through eyes, in case of an introverted person ($n_{8}$ and $n_{9}$), or in a more challenging way, both verbally and through eyes as well, in case of a more extroverted person ($n_{10}$ and $n_{11}$).

It is worth noticing that during all this, the System 2 has been waiting for information from the System 1, dynamically adapting, from a temporal perspective, the current rehabilitation plan. In the event that the System 1 detects, for example, a too high number of errors made by the user, it could communicate a “failure” feedback to KOaLa which, through a re-planning process, reconstructs a more accurate cognitive rehabilitation plan for the specific user. Similarly, if the cognitive exercise is successful, the System 1 sends a “success” feedback to KOaLa which proceeds with the execution of the current rehabilitation plan.

*User Interaction Assessment.* The example introduced in Fig. 8 was used for a preliminary evaluation of the interaction with possible real users. For this reason, the in-laboratory tests performed involved healthy people rather than users of Table 1 considered for the off-line assessment of personalization capabilities. In order to assess the usability of the system we mainly focused on the fluidity of implemented dialogues. A dialogue was considered fluid when few agent’s errors occurred and turn-taking was fairly balanced between human and artificial agent. Errors were considered when the system failed to understand the human utterance and consequently either it gave a wrong answer, or it did not answer at all. These errors often caused the human to repeat the utterance and consequently affected the length of the interaction.

***Procedure and metrics.*** Participants came to the lab and a researcher explained the experimental procedure. It was explained that some questionnaires will need to be filled in and they would have to verbally interact with a robot capable to administrate cognitive exercises. More in detail, the task consisted of participating in the “Find the word" exercise. It consisted in two sessions: a first set of simple words, and a second set of more difficult words. After the execution of the experimental task, participants were asked to fill in the *Chatbot Usability Questionnaire* (CUQ, [30]) for assessing the usability of the system. Additionally, a socio-demographic questionnaire served for collecting data about gender, age, familiarity with speech based systems and opinions about new technologies. Other metrics were collected during the experimental session to assess the dialogue performance in terms of dialogue efficiency: *length of the interaction*,* number of turns* (agent’s, user’s and total number); and dialogue quality: *errors* made by the agent [67, 68]. Since at least 30 turns (fairly balanced between user and agent) would have been necessary to complete the interaction, we considered this as the minimum amount in order to judge the task complete.

*Participants.* Ten persons participated in the testing phase. More specifically, 5 women and 5 men were involved. The mean age of the whole sample was 49.5 years old (SD= 15.61). They were asked for general information about their opinion on new technologies and about their familiarity with speech-based technology like SIRI, Ok Google, Alexa, etc. Questions were administered through a 5-point Likert scale. They reported an overall good opinion towards new technologies (M= 4.2, SD=0.63), and quite high familiarity with speech-based technology (M= 3.8, SD=0.63).

**Fig. 10:**
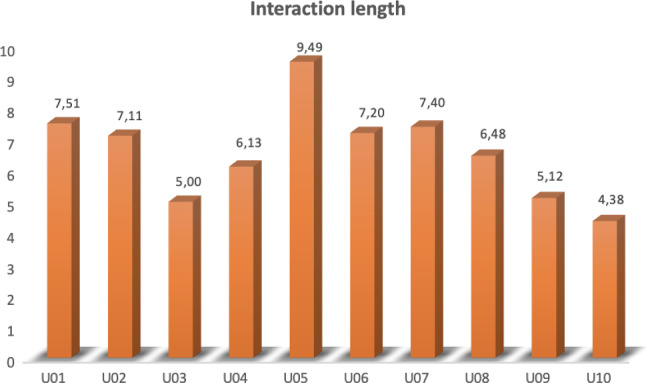
Interaction length measured in minutes for each participant

***Results*** The analysis of the performance metrics showed that the interaction with each users lasted on average 6.58 minutes (SD=1.5), with longer duration of 9.49 minutes for participant U05, and the shorter one of 4.38 minutes for participant U10 (see Fig. 10 for details).

**Fig. 11:**
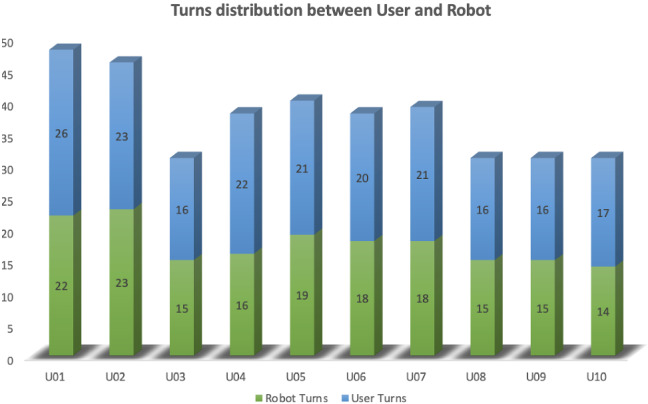
Number of users turns and robot turns per each participant

**Fig. 12:**
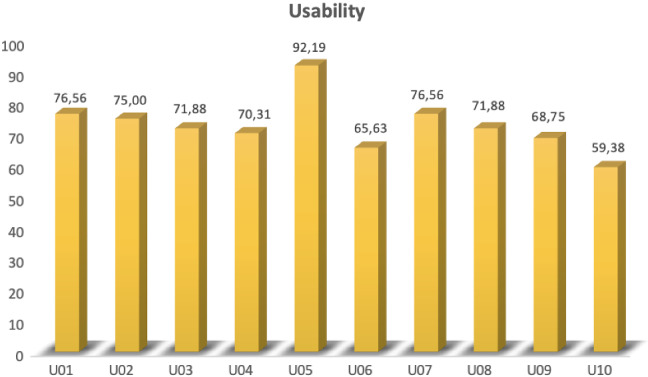
Scores obtained at the CUQ by each participant

During the interaction the robot delivered the cognitive exercise. The overall interaction consisted of an average total amount of 37.3 (SD= 6.32) turns, where the robot talked for a mean of 17.5 turns (SD= 3.1), while the user for 19.8 turns (SD= 3.45). In Fig. 11, the detailed information for each participant is reported. The slightly higher number of turns by users was mostly due to the repetition of a same request by the participants to the robot when this last one failed to answer. Nevertheless, there was a quite low amount of errors by the robot, that gave the wrong answer to a user’s question just a few times (M= 1.31, SD= 0.63), as it can be seen in Table 4 that shows how the system performed quite robustly with a low number of errors, although only in one occasion it interacted with no errors at all (User U01). The CUQ questionnaire allowed assessing the interaction usability: the results showed positive opinions regarding the interaction with the robot with a mean score of 72.81 (SD= 8.59) out of 100 (see Fig. 12 for individual scores). Overall it can be said that the usability was satisfactory and the interaction quite fluid.

**Table 4:** Frequency of robots errors during the interaction for each user

U01	U02	U03	U04	U05	U06	U07	U08	U09	U10
0	1	1	2	2	1	2	2	1	1

## Conclusions and Future Works

This article describes the research effort towards the realization of an architecture inspired by the theory of dual processes dedicated to support an intelligent and adaptive behavior of social robots that have to act with different people in dynamic environments. Based on a previous effort, the work focuses on the integration of two modules inspired by *System 1* and *System 2* of the aforementioned theory which constitute the basic modules of a mind-inspired architecture. A first module provides a long-term assistive plan, while a reactive module deals with plan execution dynamically adapting it to the contingencies of the interaction. More specifically it customizes the actions towards the user through multi-modal channels (e.g., voice, eye expression) allowing the adaptation to different affective-cognitive states.

The architecture has been exemplified in the domain of cognitive rehabilitation but both the high level plan and the adaptive actions of the robot’s behavior during execution can be generalized to other domains. An example is the domain of Human-Robot Collaboration where we have made some contribution by integrating a (slow thinking) deliberative task planning module with a (fast thinking) reactive motion planning module [18, 45]. On the one hand the task planning module is in charge of deciding the shape of collaborative plans by taking into account the capabilities of the human operator and the collaborative-robot. On the other the motion planning is in charge of “implementing" robot tasks (decided by the task planner) by dynamically adapting robot trajectories according to the observed behavior of the human operator. The work [18] in particular is an example of this kind of application. Although the work is not connected with Miriam, the presented layered architecture is coherent with the principles of the dual process theory and could be completely supported through Miriam.

Currently the two systems are implemented and integrated. An initial evaluation of the personalization capabilities of the *deliberative layer* of Miriam has been performed by taking into account real user profiles defined off-line. Furthermore, we performed laboratory experiments with healthy users on aspects related to interaction. Results show that a good level of usability of the system and a satisfactory fluidity in the dialogue. This experiment has a clear limitations that we plan to work on in the future. In fact a more robust evaluation will be designed to assess the effectiveness of the system in cognitive training with frail users with a higher number of participants as well as other aspects of the interaction.

## Data Availability

All data generated or analysed during this study are included in this published article (and its supplementary information files).

## Footnotes

1 https://www.who.int/classifications/icf/en/.

2 https://jena.apache.org/.

3 https://github.com/pstlab/PLATINUm.git.

4 We use IBM’s Watson Speech to Text service: https://www.ibm.com/cloud/watson-speech-to-text.

5 To achieve this functionality, we use the NLU module of the IBM’s Watson Assistant service: https://www.ibm.com/cloud/watson-assistant.

6 Note that “high" does not refer to some absolute numeric value. Exercises are considered as relevant if their ranking value is higher than the average value of all evaluated exercises. The dotted line of Fig. 4 shows the average ranking value computed for each user profile.

7 https://robot.omitech.it/en/sanbot-elf.

8 Developed Java-based modules can be deployed on other robotic platforms either as “stand-alone” processes interacting with ROS through services like e.g., ROSBridge — http://wiki.ros.org/rosbridge or as fully integrated ROS packages through ROSJava — http://wiki.ros.org/rosjava.
